# 

**Published:** 1893-02

**Authors:** 


### TO SUBSCRIBERS.

The periods for new subscriptions for this journal begin January and July of each year. As this is one of the periods, it may be well to inform those not familiar with the custom of this periodical that at the expiration of the subscription, a copy of the first number of the new volume is sent. If this be not returned, it is assumed that it is desired to continue the subscription. Those who wish the Journal stopped should notify the publishers at once; otherwise, they will be expected to meet the bill when presented. A careful attention to this would avoid frequent and unnecessary irritation on the part of those who have neglected a plain business requirement.

